# Supplementary material for: iGABASnFR2 is an improved genetically encoded protein sensor of GABA
Source: eLife. 2026 Mar 18;14:RP108319. doi: 10.7554/eLife.108319 (PMC12999171; doi:10.7554/eLife.108319)
Supplement: Figure 3—source data 1. [file elife-108319-fig3-data1.docx]

**Figure 3-source data 1**: Data collection and refinement statistics of iGABASnFR2 in complex with GABA

|  | iGABASnFR2+GABA |
| --- | --- |
| **Data collection** |  |
| Space group | P1 2_1_ 1 |
| Cell dimensions |  |
| *a, b, c* (Å) | 76.5, 109.9, 214.3 |
| *α, β, γ* (°) | 90.00, 100.3, 90.00 |
| Resolution (Å) | 48.74-2.60 (2.64-2.60) |
| *R_merge_* | 0.067 (0.434) |
| Mean ((I)/sd(I)) | 11.0 (2.6) |
| Completeness % | 98.5 (99.9) |
| Multiplicity | 3.6 (3.8) |
| **Refinement** |  |
| Resolution | 47.39-2.60 |
| No. reflections all/free | 105768/5396 |
| *R*_work_/*R*_free_ | 0.249/0.206 |
| No. atoms |  |
| Protein | 24386 |
| Ligand | 174 |
| Water | 32 |
| *B*-factor (Å) |  |
| Protein | 50.64 |
| Ligand | 41.32 |
| Water | 35.23 |
| R.M.S. deviations |  |
| Bond lengths (Å) | 0.007 |
| Bond angles (º) | 1.610 |
